# Competing Role of Bioactive Constituents in *Moringa oleifera* Extract and Conventional Nutrition Feed on the Performance of Cobb 500 Broilers

**DOI:** 10.1155/2015/970398

**Published:** 2015-02-22

**Authors:** Govindarajan Karthivashan, Palanisamy Arulselvan, Abd. Razak Alimon, Intan Safinar Ismail, Sharida Fakurazi

**Affiliations:** ^1^Laboratory of Vaccines and Immunotherapeutics, Institute of Bioscience, Universiti Putra Malaysia, 43400 Serdang, Selangor, Malaysia; ^2^Department of Animal Science, Faculty of Agriculture, Universiti Putra Malaysia, 43400 Selangor, Malaysia; ^3^Laboratory of Natural Products, Institute of Bioscience (IBS), Universiti Putra Malaysia (UPM), 43400 Serdang, Selangor, Malaysia; ^4^Department of Human Anatomy, Faculty of Medicine and Health Sciences, Universiti Putra Malaysia, 43400 Serdang, Selangor, Malaysia

## Abstract

The influence of *Moringa oleifera* (MO) leaf extract as a dietary supplement on the growth performance and antioxidant parameters was evaluated on broiler meat and the compounds responsible for the corresponding antioxidant activity were identified. 0.5%, 1.0%, and 1.5% w/v of MO leaf aqueous extracts (MOLE) were prepared, and nutritional feed supplemented with 0%, 0.5%, 1.0%, and 1.5% w/w of MO leaf meal (MOLM) extracts were also prepared and analysed for their *in vitro* antioxidant potential. Furthermore, the treated broiler groups (control (T1) and treatment (T2, T3, and T4)) were evaluated for performance, meat quality, and antioxidant status. The results of this study revealed that, among the broilers fed MOLM, the broilers fed 0.5% w/w MOLM (T2) exhibited enhanced meat quality and antioxidant status (*P* < 0.05). However, the antioxidant activity of the MOLE is greater than that of the MOLM. The LC-MS/MS analysis of MOLM showed high expression of isoflavones and fatty acids from soy and corn source, which antagonistically inhibit the expression of the flavonoids/phenols in the MO leaves thereby masking its antioxidant effects. Thus, altering the soy and corn gradients in conventional nutrition feed with 0.5% w/w MO leaves supplement would provide an efficient and cost-effective feed supplement.

## 1. Introduction

Poultry farming has grown since 2000, and the demand for broiler chicken meat has increased recently. From 2000 to 2010, both Africa and Asia have recorded increases of broiler meat production approximately 4.5 per cent a year, whereas growth in Europe has averaged 3.9 per cent, 3.7 per cent in Oceania and 3.5 per cent in the Americas [1]. For 2014, it is possible that the global indigenous chicken meat output will reach 95 million tons according to a forecast by the Food and Agriculture Organization (FAO). Chicken production also provides a base for the socioeconomic advancement in the majority of developing countries [2]. A primary reason for this increased demand for broiler meat is that consumers perceive that it as a healthy product that contains less fat, predominantly unsaturated fatty acids, and particularly polyunsaturated fatty acids, compared to beef or pork products [3].

The composition of the nutritional feed plays a major role in poultry meat production, which in general includes soy and corn as sources of energy and protein. In addition, the formulation of nutritional feed includes essential dietary supplements, which are important for improving the health and performance of the birds and also for enhancing the meat quality of human consumption [4]. In terms of meat quality, chicken muscle is enriched with polyunsaturated fatty acids, which is related to the increasing susceptibility of meat and meat products to fat oxidation with impaired organoleptic characteristics and decreased food shelf life [5]. Lipid peroxidation is the primary mechanism of meat decomposition and drives the necessity for antioxidant-enriched substances as an important constituent of feed supplements [6]. The advancement of poultry research has combined the knowledge of biochemical and physiological mechanisms to improve the efficiency of feed utilisation and increase desired attributes in response to changing dietary constituents [7]. Because of consumer preferences, despite the availability of synthetic antioxidants, food industries have focused on natural antioxidants as feed supplements since they are simple and cost-effective methods to achieve higher antioxidant stability, retain meat quality, and increase shelf life [8].

A major natural source of antioxidants is plants. The* Miracle Tree* or* Moringa oleifera* Lam. (MO) is postulated to have the highest antioxidant content in food and also has a remarkable range of medicinal uses and high nutritional value [9–14]. The leaves of this plant provide a rich source of carotenoids, vitamins, minerals, amino acids, alkaloids, and flavonoids and a rare combination of phenolic compounds, including zeatin, quercetin, kaempferol, apigenin, and many other phytoconstituents that offer essential and disease preventing nutrients to humans [15, 16]. MO leaf meal has been recently evaluated for its effect on chicken meat growth and quality. MO leaves incorporated into maize meal poultry feed led to better growth performance of the chickens and a significant increase in the serum level of biochemical minerals compared to the maize meal feed alone [17]. Kakengi et al. [18] reported that MO leaf meal is a rich protein source that can be used as a substitute for sunflower seed meal at up to 20 mg/kg substitution without any detrimental effects in laying chickens. MO leaf meal has also been evaluated for the replacement of antibiotics in nutritional feed supplements and the results showed that MO leaf meal was a good replacement for oxytetracycline (antibiotic) in broilers [19]. Although several studies have reported the use of MO leaves as feed supplements in livestock [20–22], the optimal concentration of MO leaves as a nutritional supplement has not yet been determined, and there are only limited reports on the bioactive constituents of MO leaves and their impact on meat antioxidant status. The food industry, poultry farmers, and researchers must determine the best concentration of MO leaf supplement for broiler production with antioxidant-enriched meat. The objective of this study is to identify the optimal concentration of MO leaves as a nutritional supplement for broilers and also to identify and evaluate the bioactive constituents of both the MO leaves and conventional feed and their impact on broiler meat antioxidant status.

## 2. Materials and Methods

### 2.1. Leaves Extract Preparation and Feed Material Formulation

Fresh and mature leaves of* M. oleifera* were harvested locally from garden-2 of Universiti Putra Malaysia and were first air-dried for 12 h at room temperature (24°C) and then oven-dried for two consecutive days, ground, and stored in air tight plastic bags until further processing. Initially, three various aqueous concentrations of MO leaf extracts (MOLE) were prepared (g leaf powder : distilled water, 0.5 g : 1 L (0.5% w/v); 1 g : 1 L (1.0% w/v); and 1.5% g : 1 L (1.5 w/v)) followed by maceration of the leaf powder in 3 different bottles for two days at room temperature. The corresponding residues were further condensed with a rotary evaporator at 40°C, freeze-dried, and stored at −80°C individually until further use. Subordinately, four concentrations of MO leaf meal (MOLM) extracts were prepared (g leaf powder : g other feed constituents, 0 g : 100 g (0% w/w); 0.5 g : 99.5 g (0.5% w/w); 1.0 g : 99 g (1.0% w/w); and 1.5 g : 98.5 g (1.5% w/w)). The MOLM feed formulations with concentrations are listed in Table 1.

### 2.2. Feeding Trial and Design

A feeding trial using Cobb 500 breed broiler chickens (23 weeks) was carried out on 160 birds retained in 6-tier battery cages (*L*: 0.5 m, *W*: 0.9 m, and *H*: 0.6 m/compartment) in a trial room with controlled climate and a light regimen of 16 h light : 8 h dark. The feed and water were provided* ad libitum*. The chickens were randomly divided into the following four groups: three treatment groups (T2 (0.5% w/w), T3 (1.0% w/w), and T4 (1.5% w/w)) and one control group (T1 (only nutritional feed)). Each group had five replicates of 8 broilers per group. The trial lasted for 42 days, and, for the first 21 days, the birds were fed a commercial starter ration, followed by the experimental diet until day 42. The experimental diet contains 20 mg crude protein (CP) and 3050 kcal metabolisable energy (ME)/kg supplemented with 0%, 0.5%, 1.0%, and 1.5% MOLM, and the chickens were fed as shown in Table 1. The chicks were inspected daily, and dead birds were removed after the mortality was recorded. The broiler chicks were weighed at the beginning of the experiment and then weekly. The feed allocation and total feed intake were also calculated weekly. The body weight (BW, g) was calculated as the final BW minus the initial BW, and the average weight gains (WG, g) were calculated accordingly. The feed intake (FI, g) was calculated as the feed allocated minus the feed refused, and the feed conversion ratio (FCR) was calculated as the FI (g) per BW (g). The feeding trial was conducted in accordance with the guidelines of the Institutional Animal Care and Use Committee, Universiti Putra Malaysia, which regulate and monitor the use and welfare of animals in experiments.

### 2.3. Sample Preparation and Analysis

On the 42nd day, the experimental birds were weighed and sacrificed using the halal procedure by severing the jugular veins. The liver, kidney, breast, and thigh muscles were carefully excised, packed in polyethylene bags, flash-frozen in liquid nitrogen, and stored at −80°C until use. The carcasses were dressed by removing the head, feet, skin, and viscera, and the dressing percentage was calculated using the formula ( = carcass weight  (CW)/live weight  (LW)∗100). The bones, muscles (meat), and fat were carefully trimmed from the carcass and weighed accordingly to calculate the meat : bone : fat ratio for each dietary treatment. Two samples from each replicate of the meat samples stored at −80°C were removed and weighed immediately (*W*_1_). The samples were then dried carefully with absorbent paper and weighed again (*W*_2_) to calculate the drip loss using the formula ( = *W*_1_ − *W*_2_/*W*_1_∗100). Two samples from each replicate of the meat samples were preweighed (*w*_1_) and then cooked in a water bath at 80°C for 20 min and cooled for 30 min to obtain the weight of the cooked sample (*w*_2_). The cooking loss was calculated with the formula ( = *w*_1_ − *w*_2_/*w*_1_∗100). The meat color and tenderness were measured on three samples from each replicate based on the L (lightness), a (redness), and b (yellowness) using the HunterLab system (Colour Flex, USA). For the measurement of meat tenderness, three cores were obtained from each sample and sheared at two locations using a texture analyser (Stable Micro Systems, UK) with a Warner-Bratzler blade. The shear force values were then reported.

### 2.4. Antioxidant Activity

A comparative analysis was performed for the* in vitro* antioxidant activities of the MOLE (0.5% w/v, 1.0% w/v, and 1.5% w/v) and MOLM (0% w/w, 0.5% w/w, 1.0% w/w, and 1.5% w/w) using DPPH radical scavenging and phosphomolybdenum total antioxidant capacity assays as described by Karthivashan et al. [9]. The liver, kidney, breast, and thigh muscles from the treated broiler chicks that were stored at −80°C were thawed, homogenized, and centrifuged, and the supernatants were assayed according to the kit instructions from the Cayman Chemical Company (Ann Arbor, U.S.A.) to determine the LPO according to standard procedures using LPO (Item number 705002) and the activity of the antioxidant enzymes SOD (Item number 706002), CAT (Item number 707002), and GPx (Item number 703102).

### 2.5. Chromatography Instrumentation and Conditions

Chromatographic separation of the compounds in the 0.5% w/v, 1.0% w/v, and 1.5% w/v aqueous MO leaf extracts (MOLE) was performed with a LUNA C18 (4 × 250 mm, 5 *μ*m) Phenomenex column (Torrance, California, U.S.A.) on an Agilent 1100 series HPLC system (Santa Clara, California, U.S.A.) equipped with a binary pump, diode array detector (DAD) (200 to 600 nm), and autosampler. The sample injection volume was 20 *μ*L with a mobile phase flow rate of 1.0 mL/min monitored at a wavelength of 254 nm. The mobile phase consisted of solvent A, distilled water, and solvent B, methanol : distilled water 70 : 30 (v/v). The gradient program profile was a combination of solvents A and B as follows: 0 to 10 min, 30% solvent B; 10 to 20 min, 40% solvent B; 20 to 35 min, 50% solvent B; 35 to 40 min, 60% solvent B; 40 to 45 min, 70% solvent B; and 45 to 50 min, 0% solvent B.

The compounds in the 0.5 w/v aqueous MO leaf extracts (MOLE) and 0.5 w/w MO leaf meal (MOLM) extracts were identified with accurate mass detection using an AB Sciex 3200 QTrap LCMS/MS with a Perkin Elmer FX 15 UHPLC system (MA, USA). The negative ion mass spectra were obtained with a LC QTrap MS/MS detector in full ion scan mode (100 to 1200 *m*/*z* for full scan and 50–1200 *m*/*z* for MS/MS scan) at a scan rate of 0.5 Hz. The system was supported with mass spectrometry software and a spectral library provided by ACD labs (Toronto, ON, Canada). Analyte separation was performed on a C18 column (4 × 250 mm, 5 *μ*m, Phenomenex) with a gradient mobile phase consisting of water (solvent A) and methanol with 1% acetonitrile (solvent B), each containing 0.1% formic acid and 5 mM ammonium format. The gradient program was 40% solvent B to 50% solvent B over 11.00 min at a flow rate of 1.0 mL/min. The sample injection volume was 20 *μ*L. All chromatographic procedures were performed at ambient temperature, and the corresponding peaks from the QTrap LCMS/MS analysis of both the 0.5 w/v MOLE and 0.5 w/w MOLM were identified by comparison with the literature/ACD labs mass spectral library.

### 2.6. Statistical Analysis

All data from the* in vitro* and* in vivo* experiments were obtained from triplicate experiments and were expressed as the mean ± S.E.M. The statistical analysis was carried out by analysis of variance (ANOVA) followed by Tukey's test using the IBM-SPSS Statistics software, version 20 (Armonk, NY, U.S.A.). The significance level was set at *P* < 0.05.

## 3. Results

### 3.1. *In Vitro* Antioxidant Activity

**Figure 1:**
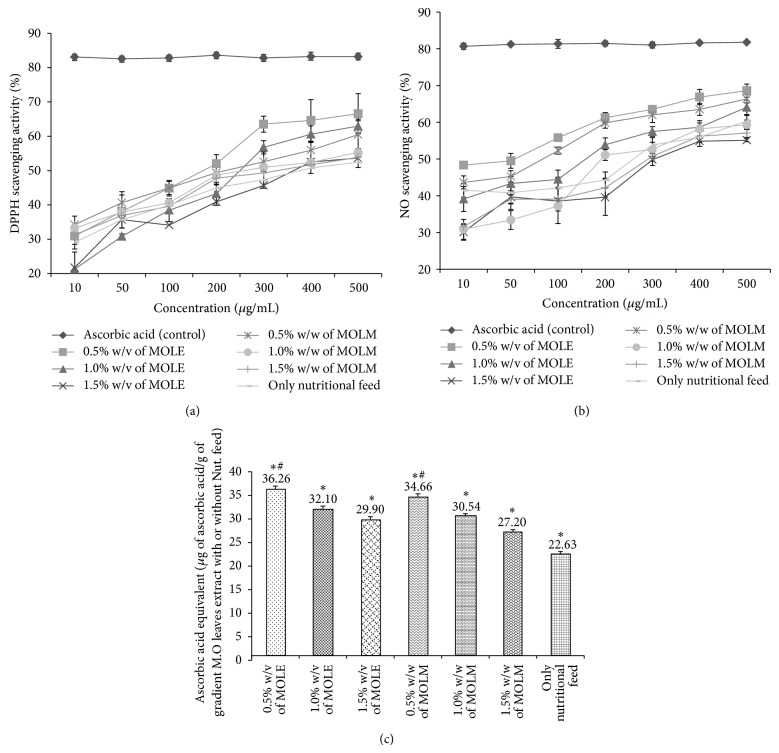
*In vitro* antioxidant activity of MOLE and MOLM extracts. (a) DPPH radical scavenging activity; (b) NO scavenging activity; and (c) total antioxidant capacity of varying gradient* Moringa oleifera* leaves extracts (MOLE) (0.5%, 1.0%, and 1.5% w/v) and* Moringa oleifera* leaves meal (MOLM) extracts (0%, 0.5%, 1.0%, and 1.5% w/w) with/without nutritional feed at different concentrations (10–500 *μ*g/mL) were determined spectrophotometrically at 540 nm, 540 nm, and 630 nm respectively. Results are means ± SD of three duplicate measurements. (DPPH: 1,1-diphenyl-2-picryl-hydrazyl free radical; NO: Nitric oxide free radical). In (a) and (b), the control compared to gradient extract with or without nutritional feed is statistically significant (*P* < 0.05). In (c), ^*^ *P* < 0.05 compared to only nutritional feed and ^#^ *P* < 0.05 compared to other gradient extracts.

The* in vitro* antioxidant activity of the MO leaf extracts (MOLE) and MO leaf meal (MOLM) extracts were comparatively evaluated with DPPH, nitric oxide (NO) radical scavenging assay, and phosphomolybdenum (PMO) total antioxidant capacity assays. For the DPPH and NO assays, the changes in the ability of the MO leaf extracts (MOLE) and MO leaf meal (MOLM) extracts to scavenge free DPPH and NO radicals were calculated as the percentage inhibition and are shown in Figures 1(a) and 1(b), respectively. The results indicate that 0.5 w/v MOLE has significant (*P* < 0.05) free radical scavenging activity, with the lowest IC_50_ values of 154.5 and 56.4 for DPPH and NO, respectively, compared to the other extracts. For the MOLM extracts, 0.5% w/w MOLM has the highest scavenging activity with IC_50_ values of 229.4 and 134.5 for DPPH and NO, respectively, but these values are not as low as the 0.5% w/v MOLE. The nutritional feed alone (0% w/w MOLM) shows the least scavenging activity with IC_50_ values of 378 and 298 for DPPH and NO, respectively, although the IC_50_ values for 1.5% w/w MOLM are higher than 0 w/w MOLM (control) for NO radical scavenging activity. For the PMO total antioxidant capacity assay, we determined whether the MO leaf extract (MOLE) and MO leaf meal (MOLM) extracts reduce phosphomolybdic acid to phosphomolybdic blue (Mo_+6_ → Mo_+5_), and this was expressed quantitatively in terms of ascorbic acid equivalent *μ*g/g of extract in Figure 1(c). The results were consistent with the DPPH and NO assay results. The 0.5% w/v MOLE had the highest antioxidant capacity (*P* < 0.05) of all concentrations, with 36.26 ascorbic acid equivalent *μ*g/g of extract. However, for the other MOLM concentration, 0.5% w/w MOLM has the highest antioxidant capacity (*P* < 0.05), with 34.66 ascorbic acid equivalent *μ*g/g of extract, and the control (0% w/w MOLM) has the lowest activity, with 22.63 ascorbic acid equivalent *μ*g/g of extract.

### 3.2. Broiler Growth Performance, Carcass, and Meat Quality

#### 3.2.1. Growth Performance

The average weight gain, feed intake (FI), feed conversion ratio (FCR), and mortality for the treatment period of 22–42 days are reported in Table 2(a). Broilers fed MOLM (T2, T3, and T4) show significant (*P* < 0.05) weight gain compared to broilers fed nutritional feed only (T1), although there were no significant differences in weight gain for the dietary treatments.

#### 3.2.2. Feed Conversion Ratio

The feed conversion ratio (FCR) data from Table 2(a) indicates that the broilers fed nutritional feed alone (T1: control group) show significantly (*P* < 0.05) highest FCR value of 2.67 ± 0.052 compared to all the dietary treatment groups (T2, T3, and T4). Throughout the experimental period, approximately 2% mortality was observed for all groups, and there were no significant differences, except for group T2 with 8% mortality, which caused the anomaly.

#### 3.2.3. Carcass Characteristics

Table 2(b) shows the average dressing percentage, meat : bone, meat : fat, and bone : fat ratio of the MOLM fed broilers. The result indicates that the broilers fed MOLM (T2, T3, and T4) have significantly (*P* < 0.05) higher dressing percentages than the broilers fed nutritional feed alone (T1: control). Of the MOLM concentrations (T2, T3, and T4), the dressing percentages were significantly (*P* < 0.05) different, ranging from 67 to 70%, and T3 (1.0% w/w MOLM) showed the highest dressing percentage of 70%. The meat : bone and meat : fat ratios were significantly (*P* < 0.05) different among the treated birds, with values ranging 3.459–3.814 and 6.31–8.43, respectively.

#### 3.2.4. Meat Quality

Table 2(c) shows the average cooking loss, drip loss, color, and tenderness of the MOLM fed broiler meat. The data in Table 2(c) indicate significant differences among all treatment groups (*P* < 0.05) for cooking loss and drip loss. The treatment groups (T2, T3, and T4) exhibited significantly higher drip loss values compared to the control group (T1), which had the lowest drip loss value. By contrast, the control group (T1), T3, and T4 exhibited the highest cooking loss without any significant difference among the groups, compared to T2 with the lowest cooking loss value of 16.62%. The percentage of cooking loss ranged from 16.62 to 21.99%. Table 2(c) shows the average color of the meat determined by the lightness (L^*^), redness (a^*^), and yellowness (b^*^) of broiler chickens fed MOLM supplements. As expected, the broiler chicken breast meat exhibited greater yellowness (b^*^) than redness (a^*^). For lightness, there was no significant difference between T2 and T4. However, T3 showed a significantly (*P* < 0.05) higher lightness value of 51.70. For redness, there was no significant difference between T1 and T4; however, T2 is significantly different (*P* < 0.05) from T3 and possesses the highest value of 7.93. Although there are slight differences among all the treatments, T4 exhibited the highest yellowness value of 14.24. Table 2(c) shows the average tenderness of broiler chicken meat fed with varying concentrations of MOLM supplement. The shear value indicates the degree of tenderness. There was no significant difference among the treatment groups; however, the T2 group showed a significantly higher value meat tenderness of 1.23 compared to T1, T3, and T4.

### 3.3. Lipid Peroxidation (MDA) Levels and Antioxidant Enzymes Activities in Chicken Muscles and Organs

**Figure 2:**
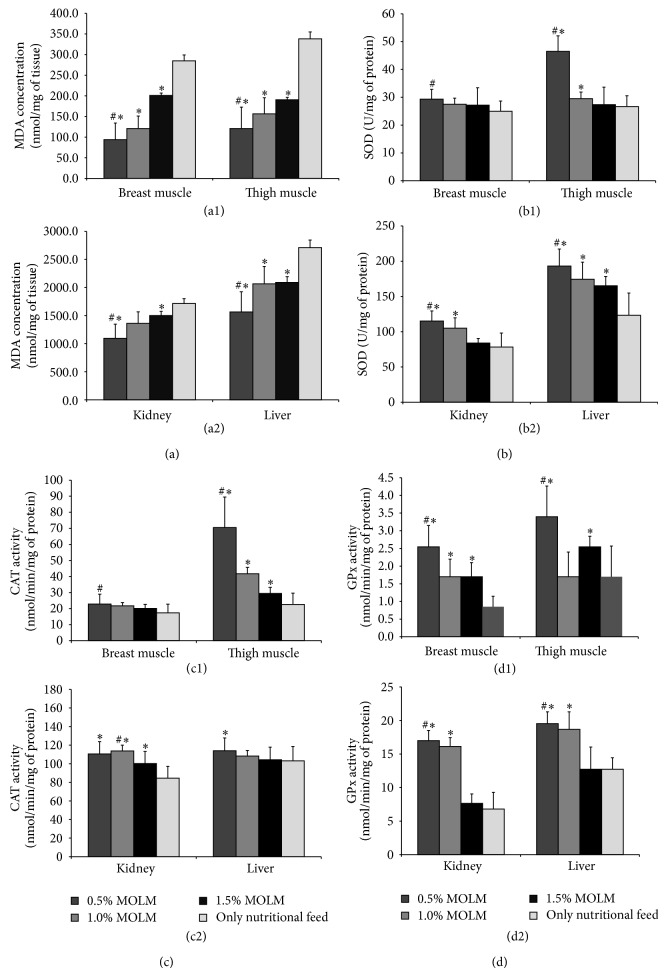
Antioxidant enzymes (MDA, SOD, CAT, and GPx) level in chicken muscles and organs. (a) Total malonaldehyde (MDA); (b) SOD activity; (c) catalase activity; and (d) glutathione peroxidase (GPx) activity of different muscles (breast and thigh) and organs (liver and kidney) of chicken fed with various gradient* Moringa oleifera* leaves extracts (0.5%, 1.0%, and 1.5% w/w) with nutritional feed/nutritional feed alone were determined. Results are means ± SD of three duplicate measurements. ^*^ *P* < 0.05 compared to only nutritional feed and ^#^ *P* < 0.05 compared to other gradient extracts.

The impact of MOLM feed supplements on the lipid peroxidation (MDA) levels in the liver, kidney, breast, and thigh muscles of treated broilers is shown in Figure 2(a). The extent of lipid peroxidation in the muscle and organ samples was measured by MDA formation. Figures 2(a)(a1) and 2(a)(a2) clearly show that the MDA formation in the breast, thigh, liver, and kidney of broilers treated with MOLM (T2, T3, and T4) were significantly (*P* < 0.05) lower than the control group (T1). The results were also significantly different among all the treatment groups (*P* < 0.05) except for kidney. Group T3 was not significantly different from the control group. However, T2 (0.5% w/w MOLM) showed the lowest MDA formation in both the muscles and organs, indicating suppressed lipid peroxidation activity. Changes in the activities of the antioxidant enzymes SOD, CAT, and GPx in the muscles (breast and thigh) and organs (liver and kidney) of broilers fed MOLM feed supplement are shown in Figure 2.  Figure 2(b)(b1) shows no significant difference in SOD levels of the broiler tissues for the MOLM treated groups and control groups. However, T2 (0.5% w/w MOLM) had the highest SOD level in both the breast and thigh muscles compared to the other treatment groups. The SOD levels in the thigh are greater than the breast tissue, particularly for T2. By contrast, Figure 2(b)(b2) shows significant differences in the SOD levels of the liver and kidney for the MOLM treated groups (T2, T3, and T4) and control group (T1). However, the SOD level in the kidney of group T3 showed was not significantly different from the control group. Group T2 (0.5% w/w MOLM) had a significantly high level of SOD in both the muscles and organs compared to the control group (T1). Figures 2(c)(c1) and 2(c)(c2) also show a similar trend because the catalase enzyme levels for T2 (0.5% w/w MOLM) in the breast, thigh, and liver were significantly higher compared to the other treatment and control groups, except for the kidney, in which T3 showed high catalase level, with a small difference for the T2 group. Although the thigh and kidney showed significant differences for all the treatment groups (*P* < 0.05) and control, no significant differences were observed in the breast and liver. Overall, T2 (0.5% w/w MOLM) showed significantly high levels of catalase in both the muscles and organs compared to the control group (T1), except the kidney of T3, possibly because of an anomaly.  Figure 2(d)(d1) shows a significant difference in the GPx activity levels of broilers tissues for the MOLM treated groups and control group, in which T2 (0.5% w/w MOLM) showed the highest GPx level in both the breast and thigh muscles compared to the other treatment groups. The thigh muscle of the T3 group (1.0% w/w MOLM) showed an unexpected drop in the GPx level, with no significant difference with the control group (T1).  Figure 2(d)(d2) shows a significant (*P* < 0.05) increase in the level of GPx activity in both the kidney and the liver of T2 (0.5% w/w MOLM) and T3 (1.0% MOLM) and a nonsignificant decrease in the GPx level in T4 (1.5% w/w MOLM) compared to the control group (T1). However, T2 (0.5% w/w MOLM) showed the highest GPx levels in both the muscles and the organs compared to the control.

### 3.4. Bioactive Constituents and Their Role in MOLE and MOLM

**Figure 3:**
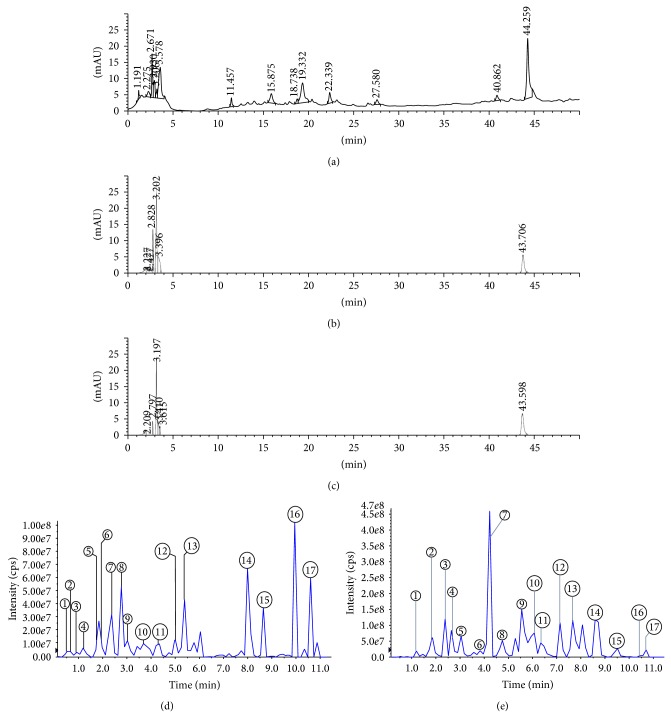
HPLC fingerprints and LC-MS/MS chromatograms. HPLC-DAD (254 nm) fingerprints of (a) 0.5%, (b) 1.0%, and (c) 1.5% w/w* Moringa oleifera* leaves extracts and LC-MS/MS (254 nm) chromatograms of (d) 0.5% w/w* Moringa oleifera* leaves extracts alone and (e) 0.5% w/w* Moringa oleifera* leaves meal with nutritional feed.

The HPLC fingerprints of 0.5% w/v, 1.0% w/v, and 1.5% w/v MOLE showed the same profile of peaks (Figures 3(a), 3(b), and 3(c)). A few peaks were uniquely expressed during 10–30 min retention time of the 0.5% w/v MOLE (Figure 3(a)), which may be responsible for its high antioxidant activity. Furthermore, LC-MS/MS analysis of the unique peaks of the 0.5% w/v MOLE identified these peaks as flavonoids with the major constituents of vitamins, carotenoids, and a few organic acids and inositol derivatives (Figure 3(d)). In addition, for comparative analysis, LC-MS/MS analysis of the 0.5% w/w MOLM extract with the same gradient program revealed the presence of isoflavones, phenols, phospholipids, and fatty acids and few flavonoids (Figure 3(e)). These compounds from the 0.5% w/v MOLE and 0.5% w/w MOLM have been tentatively identified and listed in Table 3(a) as apigenin, quercetin, kaempferol derivative, zeaxanthin, tryptophan, succinic acid conjugate, cinnamic acid conjugate, 4-p-coumaroylquinic acid, and ellagic acid conjugate and in Table 3(b) as diazin, daidzein, genistein, formononetin, ellagic acid conjugate, dimethoxyflavone conjugate, lysophosphatidylinositols, methyl stearate, linoleic acid, oleic acid, stearic acid, methyl ester fatty acids, and quercetin based on the literature [23–27]/ACD labs mass spectral library.

## 4. Discussion

### 4.1. Broiler Performance

Growth performance is a primary factor for determining the productivity of broiler chickens. The results indicate that MOLM significantly improves the growth performance of broilers, which is consistent with Ayssiwede et al. [20] who reported that* M. oleifera* leaf meal added to broiler diets significantly increased the average daily weight gain of the broilers. The feed conversion ratio is used to determine the performance of the animals and is obtained by dividing the feed intake by the weight gained. A low FCR is a good indication of high quality feed [28]. In accordance, the MOLM feed supplement increases the performance of the chickens. However, The FCR and FI results show no significant differences for the birds supplemented with MOLM which is consistent with [20]. Approximately, 2% mortality was observed among all the groups except group T2 with 8% which is considered due to anomaly as in previous studies it has been reported that the addition of MO leaf meal as a feed supplement does not produce any adverse effects on the health and mortality of broiler chickens [29]. Thus, the MO leaf meal is excluded as a factor for the raise in the mortality rate but plays a major role in improving growth performance of chicken as it is enriched with nutritional constituents.

The dressing percentage is a trait of economic importance, and the higher the dressing percentage the better the economic returns [30]. However, the results obtained were relatively less than the findings of Ayssiwede et al. [20] and Zanu et al. [29], who observed percentages ranging from 74 to 77% and 79 to 81%, respectively. This may be because of environmental and climatic variations. The water holding capacity (WHC) is the most important qualitative characteristic of meat because it affects the appearance of the product, cooking behavior, and juiciness [30]. The WHC of meat is related to the amount of free water released by the meat when subjected to physical pressure or force. The WHCs were examined in terms of drip loss, release of water from the meat without external pressure, and cooking loss, loss of water due to cooking. The treatment groups (T2, T3, and T4) exhibited higher drip loss values compared to the control group (T1), possibly due to an increase in abdominal fat yield and greater glycogen content in the meat from the broilers fed MO leaves [19]. Omojola et al. [30] stated that meat with less cooking loss would give a higher yield per unit cut. Consequently, group T2 (0.5% w/w), which had the lowest cooking loss of 16.62%, had a higher yield of broiler meat. This supports the previous results of Nkukwana et al. [22] who showed that a low percentage of leaf meal inclusion in broiler diets significantly improves broiler performance. The color of raw poultry meat is critical for consumer selection, and the major contributing factors to poultry meat color are myoglobin content, meat pH, intramuscular fat, and moisture content. However, the lightness of broiler chicken meat is more significant than the other colors because it is 100% white fibers and is the most preferable because of its attractiveness and acceptance among consumers. Accordingly, the results indicate that the treatment groups have significantly higher meat lightness compared to the control group (T1), possibly due to the beta-carotene consumed by chickens fed the MOLM supplement [31].

Meat tenderness is a function of collagen content, heat stability, and myofibrillar structure of the muscle. Meat tenderness significantly improves with muscle aging because of the breakdown of myofibrillar proteins. Tenderness is an important attribute that consumers consider when purchasing chicken meat [32]. The shear value indicates the degree of tenderness. The MO leaves treated chickens exhibited good meat tenderness, especially group T2 (0.5% w/w). This is consistent with the results of Muchenje et al. [33], who reported that when an animal is given supplementary feed, it accrues more intramuscular fat than the one that is not provided supplemental feed. Meat juiciness is directly related to the intramuscular lipids and moisture content of meat and improves tenderness.

Cumulatively, the results of the growth performance, carcass, and meat quality assays indicate that broilers fed MOLM feed supplement show significant (*P* < 0.05) improvement in health status, performance, and meat quality compared to the control group fed only conventional feed. However, consistently varying results (i.e., T4 exhibiting low FCR with high performance; T3 exhibiting improvement in carcass yield; and T2 exhibiting high meat tenderness) were obtained for the MOLM treated groups (T2, T3, and T4). This makes it difficult to select the optimal concentration of MOLM based on the growth performance, carcass, and meat quality. However, 0.5% w/w MOLM fed group retained the meat tenderness, which is an important factor for commercialization. Furthermore, the efficiency of the MO supplement was evaluated.

### 4.2. Antioxidant Analysis

The results of the* in vitro* antioxidant activity assays indicate that the MOLE extracts show high radical scavenging activity and total antioxidant capacity compared to the MOLM extracts. The order of antioxidant activity/capacity was as follows: 0.5% w/v MOLE > 0.5% w/w MOLM > 1.0% w/v MOLE > 1.0% w/w MOLM > 1.5% w/v MOLE > 1.5% w/w MOLM > 0% w/w MOLM (only nutritional feed). These results support a previous report indicating aqueous MOLE exhibit high antioxidant activity that may be attributed to phytoconstituents, such as polyphenols, tannins, anthocyanin, glycosides, and thiocarbamates that scavenge free radicals, activate antioxidant enzymes, and inhibit oxidases [34]. In the form of feed supplement (MOLM), the efficiency of the MO leaf extract is lower than the leaf extract alone; however, compared to the nutritional feed alone, the MOLM shows higher activity, supporting the results of Liu et al. [35] and Eloff [36]. In addition, chickens do not voluntarily consume MO leaves; therefore, MOLM can be used as a feed supplement to increase broiler performance [37]. However, based on these evidences, among the evaluated concentrations, 0.5% w/w MOLM shows the high antioxidant activity due to enriched antioxidant supplement from MO leaves.

In accordance with the* in vitro* results, 0.5 w/w MOLM exhibited lowest MDA level and highest SOD, CAT, and GPx activity in both liver and tissue. This is due to the MOLM supplement, enriched with phenolics, flavonoids, and vitamin C, inhibits the formation of free radicals, and contributes to the retention of muscles and organs and lipid stabilization. This result is consistent with the data for meat quality improvement, in which the T2 group (0.5% w/w) has a significantly higher meat tenderness value compared to the other groups. Therefore, the addition of 0.5% w/w MO with conventional feed (MOLM) retains meat quality by reducing the activity of lipid peroxidation.

Overall, the results indicated that broilers fed the MOLM supplement have significantly better performance and antioxidant status compared to the control fed only nutritional feed. For the percentage of MOLM added to the conventional meal, although the results of the growth performance and carcass and meat quality show some inconsistencies, the T2 (0.5% w/w MOLM) group showed significantly higher* in vitro* and* in vivo* antioxidant activity, which is consistent with increased meat quality.

### 4.3. The Antagonistic Behavior of Bioactive Constituents between MOLE and MOLM

Though antioxidant assay results convincingly revealed 0.5% w/w MOLM as the optimal gradient for feed supplement, the* in vitro* results specified that generally MOLE exhibit higher antioxidant activity than MOLM, and this drives us to analyze the role of constituents in the activity. In accordance, firstly the comparative analysis of the 0.5% w/v, 1.0% w/v, and 1.5% w/v MOLE using HPLC revealed that 0.5% w/v exhibit few unique peaks further confirmed as flavonoids using LC-MS/MS. In the previous study, the presence of apigenin, quercetin, and kaempferol has been reported in* M. oleifera* leaves, which are responsible for its antioxidant activity [9]. This was also consistent with results of a study by Siddhuraju and Becker [15]. Thus, MOLE are enriched with antioxidant boosting flavonoids.

Secondly, LC-MS/MS was further used to compare and identify compounds in the 0.5% w/v MOLE and 0.5% w/w MOLM (T2) and their role in meat antioxidant status. As anticipated, 0.5% w/v MOLE exhibited high expression of flavonoids. Previously Gupta et al. [38] isolated quercetin and kaempferol from* M. oleifera* leaf extract and identified their antidiabetic property as restoring the antioxidant status in streptozotocin-induced diabetic rats. Qwele [39] also reported the presence of zeaxanthin and tryptophan in* M. oleifera* leaves and categorized them as antioxidant compounds because they enhance endogenous antioxidant enzyme levels. Coppin [40] reported the presence of coumaroylquinic acid, and Sinha et al., 2012 [41], reported the presence of ellagic acid conjugate in* M. oleifera* leaves, which may prevent hepatic lipid peroxidation by scavenging free radicals. Together, these results indicate that 0.5% w/v MOLE are enriched with antioxidant compounds, such as flavonoids, vitamins, carotenoids, and a few organic acids, which may be responsible for its enhanced antioxidant activity.

On contrast, 0.5% w/w MOLM possesses a high amount of isoflavones, phospholipids, and fatty acids, possibly from the soy and corn ingredients of feed. In a previous study, He and Chen [42] reported the occurrence of the isoflavone diazin and its aglycones, daidzein, genistein, and formononetin in soybeans. This clearly indicates that the isoflavones in the 0.5 w/w MOLM supplement may be from the soy in the conventional feed mix. These compounds exert antioxidant and anticarcinogenic effects in the skin of hairless mice and protect cells against oxidative DNA damage [43]. However, these isoflavones exhibit less potent antioxidant activity than flavonoids and also show antagonism against the antioxidant activity of flavonoids, as reported by Choi et al. [44]. The chromatographic analysis also showed high level expression of fatty acids, such as linoleic acid, oleic acid, stearic acid, and methyl ester fatty acids, which are expressed in* M. oleifera* leaves, soy, and corn and are consistent with a previous report [45]. The LC MS/MS analysis results clearly support the antioxidant results, in which MOLE have high antioxidant activity because of the high expression of flavonoids, which was suppressed in MOLM because of the antagonistic role of isoflavones (from conventional feed) against flavonoids (from MO leaves). However, MOLM exhibits less potent antioxidant activity because of the presence of isoflavones, quercetin, and fatty acids.

## 5. Conclusion

Conclusively, during MOLM preparation, the compounds in the conventional feed, such as the isoflavones, phospholipids, and fatty acids, antagonistically inhibit the expression/activity of the flavonoids and phenols in the MO leaves. Therefore, 0.5% w/w MOLM showed relatively low antioxidant activity compared to the 0.5% w/v MOLE. However, isoflavones, phospholipids, and fatty acids also possess antioxidant effects, relatively less than flavonoids. Consequently, the chickens fed 0.5% w/w MOLM feed supplement showed the highest antioxidant activity compared to the other treatment and control groups. Thus, optimizing the gradient of soy and corn in the conventional nutrition feed with 0.5% w/w of MOLE might help the researchers/nutritional feed corps/farmers to provide an efficient and cost-effective feed for broilers.

## Figures and Tables

**Table 1:** Composition of experimental diet.

Feed stuff ingredients (%)	Control	0.5% MOLM	1.0% MOLM	1.5% MOLM
	T1	T2	T3	T4
*Moringa oleifera* leaves meal	0	0.5	1.0	1.5
Corn	61	60.5	60.5	60.5
Soybean meal	27	27	26.5	26
Fish meal	5	5	5	5
Palm oil	4	4	4	4
Salt	0.25	0.25	0.25	0.25
Limestone	1	1.0	1.0	1.0
Dicalcium phosphate	0.5	0.5	0.5	0.5
Minerals	0.25	0.25	0.25	0.25
Vitamins	0.25	0.25	0.25	0.25
DL-Methionine	0.15	0.15	0.15	0.15
Lysine	0.5	0.5	0.5	0.5
Choline chloride	0.1	0.1	0.1	0.1
Total	**100**	**100**	**100**	**100**

**Table:** (a) Growth performance

Parameters	Treatment groups			
	Nutritional feed only (T1)	0.5% w/w of MOLM (T2)	1.0% w/w of MOLM (T3)	1.5% w/w of MOLM (T4)
Final body weight (g) 42 d	2091 ± 45.9^b^	2222 ± 25.3^a^	2263 ± 30.2^a^	2218 ± 47.6^a^
Initial body weight (g) 42 d	707 ± 4.38^a^	705 ± 6.09^a^	702 ± 4.68^a^	701 ± 5.81^a^
Weight gain (g/day) 22–42 d	66 ± 1.986^b^	72 ± 1.285^a^	75 ± 1.439^a^	72 ± 2.409^a^
Feed intake (g/day) 22–42 d	175 ± 2.05^a^	178 ± 0.85^a^	174 ± 1.4^a^	165 ± 1.16^b^
FCR 22–42 d	2.67 ± 0.052^a^	2.47 ± 0.037^b^	2.32 ± 0.027^bc^	2.30 ± 0.065^c^
Mortality (%) 22–42 d	3 ± 2.5	8 ± 5.0	2 ± 2.5	3 ± 2.5

**Table:** (b) Carcass characteristics

Parameters	Treatment groups			
	Nutritional feed only (T1)	0.5% w/w of MOLM (T2)	1.0% w/w of MOLM (T3)	1.5% w/w of MOLM (T4)
Dressing percentage	65.97 ± 0.074^d^	67.07 ± 0.042^c^	70.15 ± 0.219^a^	68.56 ± 0.114^b^
Meat : bone	3.459 ± 0.016^b^	3.412 ± 0.024^b^	3.814 ± 0.027^a^	3.712 ± 0.050^a^
Meat : fat	6.31 ± 0.016^d^	7.89 ± 0.030^b^	8.43 ± 0.045^b^	6.83 ± 0.040^c^
Bone : fat	3.91 ± 1.13^b^	3.65 ± 1.16^a^	3.54 ± 0.51^a^	3.79 ± 0.46^a^

**Table:** (c) Meat quality

Parameters	Treatment groups			
	Nutritional feed only (T1)	0.5% w/w of MOLM (T2)	1.0% w/w of MOLM (T3)	1.5% w/w of MOLM (T4)
Cooking loss (%)	21.99 ± 0.464^a^	16.62 ± 0.619^b^	21.66 ± 1.441^a^	20.34 ± 1.141^a^
Drip loss (%)	5.72 ± 0.027^d^	5.94 ± 0.028^c^	8.01 ± 0.034^a^	7.74 ± 0.065^b^
Lightness	46.70 ± 1.193^b^	46.24 ± 0.373^b^	51.70 ± 0.827^a^	47.25 ± 0.513^b^
Redness	6.15 ± 0.583^ab^	7.93 ± 0.793^a^	5.45 ± 0.450^b^	6.64 ± 0.497^ab^
Yellowness	12.08 ± 0.604^b^	13.52 ± 0.384^ab^	13.15 ± 0.537^ab^	14.24 ± 0.584^a^
Tenderness	1.07 ± 0.1^a^	1.23 ± 0.19^a^	0.94 ± 0.07^a^	0.89 ± 0.007^a^

Table 2(a) (growth performance): the final body weight, weight gain, feed intake, feed conversion ratio, and mortality (^*^a, b values for final body weight, weight gain, and feed intake showing different superscripts within rows are significantly different (*P* < 0.05) and the ^*^a, b, bc, and c within rows of the FCR are significantly different (*P* < 0.05)). Table 2(b) (carcass characteristics): the dressing percentage and meat : bone : fat ratio (^*^a, b, c, and d values for dressing percentage and meat : bone : fat ratio within rows are significantly different (*P* < 0.05)). Table 2(c) (meat quality): the cooking loss, drip loss, color, and tenderness (^*^a, b, c, d values with different superscripts on the same row are significantly different (*P* < 0.05)), of broiler chicken fed with various gradients of *Moringa oleifera* leaves meal (0.5%, 1.0%, and 1.5% w/w) MOLM with/without nutritional feed.

**Table:** (a) Compounds identified from the chromatogram of 0.5% w/w *Moringa oleifera* leaves extracts

Peak	Retention time (RT)	Molecular ion peak (M–H)^−^	MS^2^ fragment ions intensity	Tentative compounds identified
1	0.52	117	73 (100), 99, 72	Succinic acid conjugate
2	0.65	164	147 (100), 119, 103, 72	Cinnamic acid conjugate
3	0.92	203	116 (100), 142, 159	Tryptophan
4	1.19	337	191, 163, 119 (100)	4-p-Coumaroylquinic acid
5	1.85	593	383, 353 (100), 325, 297	Apigenin-6,8-di-C-*β*-D-glucopyranoside
6	2.24	432	284 (100), 283, 312, 269	Apigenin 6 C glucoside (isovitexin)
7	2.36	252	208, 151, 164, 107 (100)	Zeaxanthin derivative
8	2.78	266	222, 178, 170 (100), 151	Unknown
9	3.04	301	257, 108 (100), 109, 65	Quercetin
10	3.73	330	294, 230, 224, 212 (100)	3,30-di-O-Methyl ellagic acid conjugate
11	4.31	309	291, 263, 251, 247, 211, 197 (100), 171	Unknown
12	5.03	481	283, 255 (100), 224, 168, 153	Kaempferol derivative
13	5.42	480	285, 255 (100), 242, 224, 168	Kaempferol derivative
14	8.07	856	596, 575, 431, 297, 279 (100), 241, 153	Inositol derivatives
15	8.72	739	279 (100), 241, 153	
16	10.04	858	279 (100), 241, 153	
17	10.7	832	277, 255 (100), 241, 153	

**Table:** (b) Compounds identified from the chromatogram of 0.5% w/w *Moringa oleifera* leaves extracts with nutritional feed

Peak	Retention time (RT)	Molecular ion peak (M–H)^−^	MS^2^ fragment ions intensity	Tentative compounds identified
1	1.18	253	223, 180, 81 (100), 80	Diazin
2	1.85	253	223 (100), 208, 180, 132, 81	Daidzein
3	2.37	269	159, 133, 65 (100), 63	Genistein
4	2.65	253	223 (100), 208, 180, 167, 133	Formononetin
5	3.00	269	159, 134, 65 (100), 63	Genistein
6	3.83	376	296, 278, 192, 133, 80 (100)	Unknown
7	4.22	393	329 (100), 301, 224, 209, 183	Ellagic acid conjugate
8	4.75	313	295, 283, 201, 183 (100)	Dimethoxyflavone conjugate
9	5.57	295	277 (100), 233, 183, 195, 171	Unknown
10	6.08	572	315, 255 (100), 241, 153	Lysophosphatidylinositols
11	6.38	299	297, 282 (100), 255	Methyl stearate
12	7.14	280	279 (100), 261, 244	Linoleic acid
13	7.68	281	256 (100), 255	Oleic acid
14	8.65	283	281 (100), 253	Stearic acid
15	9.5	382	311 (100), 255, 171, 125	Methyl ester fatty acids
16	10.5	364	301, 255, 171 (100)	Quercetin
17	10.7	384	311 (100), 255, 169, 125	Methyl ester fatty acids
